# Phytochemical variations antioxidant, and antibacterial activities among zebaria sumac (*Rhus coriaria* var. *zebaria*) populations in Iraq

**DOI:** 10.1038/s41598-024-53635-7

**Published:** 2024-02-27

**Authors:** Saba Shahrivari, Saeed Mizgeen Saeed Zeebaree, Saeideh Alizadeh-Salteh, Hadar S. Feizy, Mohammad Reza Morshedloo

**Affiliations:** 1 Department of Analytical Chemistry, Faculty of Pharmacy, Medical University of Gdansk, Gdansk, Poland; 2 Department of Horticultural Sciences, Faculty of Agriculture, University of Tabriz, Tabriz, Iran; 3 Department of Recreation and Ecotourism, College of Agricultural Engineering Sciences, University of Duhok, Duhok, Iraq; 4 Department of Horticultural Sciences, Faculty of Agriculture, University of Maragheh, Maragheh, Iran

**Keywords:** Antibacterial, Antioxidant, Essential oil, Flavonoid, *Rhus coriaria*, Sumac, Biochemistry, Microbiology, Plant sciences

## Abstract

Sumac (*Rhus coriaria* L.) is one of the medicinal plants of Anacardiaceae family and widely used as a spice in Iran and Arab countries. *Rhus coriaria* var. *zebaria* is a small tree or large shrub, wildly growing in Iraq and described as a new variety with special characteristics. These increase the importance of studying sumac in these areas. Here, the phytochemical variations and the antibacterial activity of 50 accessions of this variety from five different climatic conditions was evaluated in order to identify the best accession to use and the best area for its cultivation. This is the most comprehensive study on this plant. Essential oil compounds were identified using GC–MS method and according to the results, Z, E-2,13-octadecadien, caryophyllene oxide, 2,4-decadienal, E-caryophyllene and nonanoic acid were among the main compounds. Also, the variety is a rich source of minerals including K, Ca, Mg, Na, P, and N. Sumac fruit extract from Akre Xerds had the highest anthocyanin and the lowest amount was from Kavilca region. The radical scavenging effect of extract from Dostic area in the concentration of 400 µg/mL is closer to the effect of ascorbic acid. The largest inhibition was found in the sumac extracted oil of Xasto Zhere area against *S. aureus* in compared with penicillin and amoxicillin and enrofloxacin antibiotics.

## Introduction

Sumac (*Rhus coriaria* L.) is the only indigenous species of the *Rhus* genus in Iraq. The natural distribution of the genus is in the Mediterranean area and North Africa, as well as in Iran, Caucasus and Central Asia^1^. About the root of word, someone thinks it has been derived from "Sumaga" in Syrian, which often means "red"^2^. It is rich in tannin and in traditional medicine is used as an astringent and blood fat controller. Also, sumac is used to avoid the occurrence of pox in the eye^3^, and in Iran is traditionally used as a food additive to give acidic and sour flavor to foods. Sumac has many other therapeutic effects, such as decreasing fever and cleaning the digestive tract^4^. Due to the increase in human population and the change in the earth's climate, studies continue to find plants that meet the nutritional and medicinal needs of humans and require little fertilizer and irrigation. Sumac can also be grown in areas that are not agriculturally suitable for many plants, and its various species are used by people for different purposes; it shows the potential of these plants to commercialize their bioactivity without competing in land use for food production^5^. In addition, due to the increasing use of sumac in various industries and veterinary practices has an economic importance^6^. *Rhus coriaria* L. has a reddish-brown color, but *Rhus coriaria* var. *zebaria* has a dull whitish brown color^7^.

Phytochemical experiments on sumac have shown that its leaves and fruits are a rich source of tannins, flavone, anthocyanins and organic acids. About sumac fruits and leaves, a significant potential for antimicrobial and antioxidant effects has been reported^4^. Also, ripe fruits of sumac are rich of vitamins, amino acids and malic acid^8^.

Essential oils are among the important secondary metabolites of medicinal and aromatic plants. They have many usages in various industries and fields; such as pharmaceutical, cosmetic, food and aromatherapy industries^9^. Also, minerals are important biochemical elements that, in case of deficiency, cause symptoms and problems in human health^6^. Actually, some factors, such as geographic and environmental conditions can affect the amount of essential oils and minerals of plants.

Escherichia coli is, paradoxically, both the most frequent commensal aero-anaerobic Gram-negative bacillus of the vertebrate gut and one of the main pathogens, being responsible for both extraintestinal and intraintestinal infections. In a single individual there are resident and transient strains and also predominant strains. Clones, which are characterized by their phylogenetic group, are distributed according to environmental factors and the gut morphology, diet and body mass of hosts^10^. In the other hands, staphylococci are Gram-positive bacteria and they characterized by individual cocci. Many species of Staphylococcus genus preferentially colonies the human body. Most species require an organic source of nitrogen, supplied by 5 to 12 essential amino acids, such as valine, arginine, and B vitamins, including thiamine and nicotinamide^11^. These things attract the attention of researchers to study the effect of herbs on these bacteria.

In this study, zebaria sumac fruits were collected from different regions of Duhok province, Iraq, and their chemical compositions of essential oils, TPC, TFC and antioxidant activity of extracts, antibacterial properties of fruit oil, fruit minerals, acidity, crude proteins, total carbohydrate, crude oil and wax were investigated and compared in order to obtain relatively complete information about this newly known variety and pave the way for its use in the food and pharmaceutical industries.

## Material and methods

### Chemicals and reagents

DPPH was purchased from Sigma–Aldrich company (MO, USA). Other chemicals and solvents were analytical grade and were purchased from Merck (Darmstadt, Germany).

### Plant materials, extraction and analysis of essential oil

*Rhus coriaria* var. *zebaria* accessions were collected from different cities of Duhok province, and identified by Department of Recreation and Ecotourism, University of Duhok. All accessions were obtained under national and international guidelines and the plants were collected under the supervision and permission of Duhok University and all authors comply with all the local and national guidelines. The voucher specimen was deposited in the herbarium of Horticultural department, Duhok University, Iraq (No: 2885).

Fruits and leaves of sumac accessions were collected from Hashtka (Dinarta area, 36.785676° N, 43.931934° E, No: 2885), Xerds (Akre city, 36.805621° N, 43.843882° E, No: 3477), Dustak (Zebari area, 36.862403° N, 43.881755° E, No: 3564), Kavilca (Zebari area, 36.863599° N, 43.881733° E, No: 3565) and Xasto Zheri (Sheladze area, 36.985238° N, 43.794057° E, No: 3031). Then collected plant materials putted in a polyethylene bag to prevent loss of moisture during transportation in the field to the laboratory. Collected fruit and leaf naturally dried in the shade condition and they were powdered using an electric home blender. The powdered samples were taken to the place of testing in a polyethylene bag.

One hundred grams of powdered plant was used to extract essential oil by water distillation method. Water to plant material ratio in the process was 5:1 and the duration of extracting was 3 h.

The GC–MS analysis was performed on an Agilent 6890N-5973A (USA) with HP-5MS column. Ionization voltage was 70 electron volts. The scanning range of the spectra was set from 50 to 450 m/z. The initial temperature of the oven was 50 °C and stopped at that temperature for 5 min. Thermal gradient was 3 °C per minute and the temperature was increased to 240 °C, and then stopped for 10 min at that temperature. For each sample 1 µL was injected to the instrument. The split ratio was 1:34. Helium gas was used as a carrier gas with a flow rate of 1 ml per minute.

Identification of compounds was done by injection of valid standards (Sigma – Aldrich, USA and also by using computer library (WILEY275, NIST 05, ADAMS). Quantification was also done by the GC-FID device.

### Total phenolic content (TPC) analysis

The method of Alhakmani et al.^12^ was applied to measure total phenolic content, using the Folin-ciocalteu regent. Methanolic extract (15:50) concentrated at 40 °C by rotary vacuum evaporator. Then 1 ml of concentrated extract material solution was mixed with 1 mL of Folin-Ciocalteu’s phenol reagent in a test tube. The mixture was kept in dark about 5 min and added 10 ml of 7% sodium carbonate (Na_2_CO_3_) solution to the mixture followed by the addition of 13 ml of deionized distilled water and mixture was shaken thoroughly until mixed completely. For completion of the reaction, the mixture was kept in the dark for about 30 min at room temperature 20–23 °C, after which the absorbance of the blue color of the mixture was measured at 760 nm using spectrophotometer (Shimadzu UV-1800 UV/Visible Scanning Spectrophotomete, Japan). The total phenolic content was determined by standard curve of Gallic acid solution and the results were expressed as milligram of gallic acid equivalents (mg GAE/100 g) of extracted dried sample.

### Determination of total flavonoid content (TFC)

The method of Saeed et al.^13^ was used for determination of TFC. In this method, 0.5 ml of methanolic extract was mixed with 1.5 ml of methanol, 0.1 ml of 10% aluminum chloride, and 0.1 ml of potassium acetate (mol/l) and 2.8 ml of distilled water. The mixture remained at room temperature for 30 min and then, the absorbance was read at 415 nm with spectrophotometer spectrophotometer (Shimadzu UV-1800 UV/Visible Scanning Spectrophotomete, Japan). The same procedure was applied to all standard quercetin solutions (12.5–100 μg /ml) in methanol and a standard curve was obtained. Results were expressed as milligrams of quercetin equivalent per gram (mg QE/g) of extract.

### Determination of the total anthocyanin content of the extracts

Total anthocyanin was estimated by a pH differential method using spectrophotometer. Absorbance was measured at 510 nm and 700 nm in buffers at pH = 1 and pH = 4.5, and using a molar extinction coefficient of 29.600. Results were expressed as mg cyanidin-3-glucoside equivalent per g dry extract equation^14^.

$$ {\text{A }} = \, \left( {{\text{A51}}0{-}{\text{A 7}}00} \right){\text{ PH 1}}.0{-}\left( {{\text{A51}}0{-}{\text{A 7}}00} \right){\text{ PH 4}}.{5,} $$

$$ {\text{Total anthocyanin Conc}}. \, = {\text{ A }} \times { 29}.{6}00. $$

A: Absorbance.

### Antioxidant activity of sumac extracts (DPPH)

Matthaus^15^ method was used to evaluate the antioxidant activity. Different levels of methanolic extracts (50, 100, 200, 400 and 800 μg/ml) were reacted with 0.2 ml of DPPH (50 mg of DPPH in 100 ml methanol) or ascorbic acid as standard. The mixture was carried to a total volume of 4.0 ml by the extraction solvent. After keeping the mixture in the dark for 30 min, absorbance was read against the blank at 515 nm.

### Total acidity (%)

Total acidity was determined in the fruit juice (as malic acid) because malic acid has identified as the cause of sour taste in sumac. In this test, 0.1 N NaOH (until pH 8.1) was used and phenolphthalein was used as an indicator^16^.

### pH

To determine the rate of pH level in *Rhus coriaria* var. *zebaria* 5% w/v aqueous solution was prepared and the pH of solutions was determined using a calibrated digital pH meter (JENWAY- 3510)^17^. Standard solutions with pH 4, 7 and 9 were used.

### Fruit minerals content (N, P, K, Na, Ca, Mg, Cl)

To determine nutrients, 0.5 g of powdered dry fruits dissolved in 10 ml of H_2_SO_4_ (97–99%) was used. To clarify the color, 2 ml of perchloric acid was used and the volume was increased to 50 ml by distilled water.

#### Nitrogen (%) in fruit

The percentage of total nitrogen was obtained according to the method of Estefan et al. through the automatic Kjeldahl distillation system^18^.

#### Phosphorus (%) in fruit

It was determined according to colorimetric method using Spectrophotometer^18^.

#### Potassium and sodium (%) in fruit

They were determined via the flame photometer^19^.

#### Calcium and magnesium (%) in fruit

EDTA was used to measure calcium percentage^20^.

#### Chloride (%) in fruit

According to the Mohr method, chromate ions were used as indicator in the titration of chloride ions with a silver nitrate standard solution. After the formation of chromate deposit, the experiment finished.

### Determination of crude oil and wax in fruit

Crude oil and wax of powdered plants were extracted by soxhlet method for 6 h. using diethyl ether solvent. Powdered fruits with different weights (155.32, 113.48, 120.36, 194.85, and 83.58 g), 150 ml of 99.5% diethyl ether and temperature 35–45 were used. After the solvent evaporated and the flask cooled, it was weighed again^21^. The percentage of crude oil and wax producer was calculated using the formula:

$$ {\text{Crude oil }}\left( \% \right) \, = \, \left[ {{\text{W}}_{{{\text{oil}}}} \left( {{\text{gr}}} \right)/{\text{W}}_{{\text{original sample}}} \left( {{\text{gr}}} \right)} \right] \, \times {1}00, $$

$$ {\text{Crude wax }}\left( \% \right) \, = \, \left[ {{\text{W}}_{{{\text{wax}}}} \left( {{\text{gr}}} \right)/{\text{W}}_{{\text{original sample}}} \left( {{\text{gr}}} \right)} \right] \, \times {1}00. $$

### Determination of crude proteins (Kjeldahl method)

Kjeldahl method, which includes protein digestion, distillation and titration, was used to determination of crude protein^22^.

#### Protein digestion

The protein content was determined by digesting 0.5 g of sumac bulbs and leaves with 10 ml of 98%concentrated H_2_SO_4_ and adding 2 ml 70%perchloride acid before heating. After cooling, they were increased to 100 ml with distilled water.

#### Protein distillation

The Markham distillation unit and 5 ml of 2% acid boric were used. Then, 5 ml of the digested sample was dissolved in 5 ml of (10 N) NaOH and steamed until enough ammonium salt was collected.

#### Protein titration

The solution in the receiving flask was titrated with 0.01 N HCl until change color to a red endpoint and was determined by estimating the amount of the HCl used in the reaction. After titration, the percentage of nitrogen was calculated using the following formula:

$$ \% {\text{N}} = \left[ {{\text{V}}_{{{\text{HCl}}}} \times {\text{N}}_{{{\text{HCl}}}} \left( {0.0{1}} \right) \times {\text{Nitrogen atomic mass }}\left( {{14}} \right)/{1}000} \right] \times \left[ {{1}00{\text{ ml D}}.{\text{ water}}/{\text{5 ml Sample solution}}} \right] \times \left[ {\% {1}00/0.{\text{5 W}}_{{{\text{sample}}}} } \right], $$

V_HCl_: Volume (ml) of acid required to titrate, N_HCl_: Normality of the HCl, D. water: Distilled water, W_sample_: Weight of a sample (g), % Crude protein = %N˟F (6.25), F = Conversion factor is equivalent to (6.25).

### Total carbohydrate (%)

After combining 1 ml of the sumac fruit juice, 1 ml of 5% phenol and 18 ml of distilled water, 5 ml of H_2_SO_4_ (97%) was added^23^. After that the mixture was put in the water bath on 60 ºC for 30 min. Then 10 ml of solution was taken into the centrifuge tube at (3000 r/min.) for 15 min. Finally, the temperature of the sample reached room temperature, the total sugar was calculated at 488 nm using a spectrophotometer^24^.

### Antibacterial properties of sumac oil

Sumac oil and water extract were used to assay antibacterial properties. Soxhlet method was used to extract the oil and NaOH was used to reduce the pH of the extract. Fresh bacteria cultures (24 h) were kept in the refrigerator until use. The pH considered for the growth of *E. coli* bacteria was 6.5–7.5 and for Staph bacteria was 6–7. The water extract was passed through a filter paper before reducing the nutrients or pH. The agar well diffusion method was used to evaluate the antibacterial activities of *Rhus coriaria* var. *zebaria* against gram positive and negative bacteria^25^. The sterile micropipette was used for tacking 100 μL of prepared culture were spread on Mueller–Hinton agar, and 100 μL of each extract were directly impregnated into the wells of the agar plates by using sterilized cotton swabs. In this experiment, agar disk-diffusion method of Balouiri et al.^26^ was applied and water extract of sumac was considered as negative control and penicillin (6 μg), enrofloxacin (5 μg), amoxicillin (25 μg), and florfenicol (30 μg) antibiotic disks (Conda lab, Spain's) were used as positive controls. After 24 h of incubation at 37˚C the diameter of the inhibition zone was measured^26^.

## Results and discussion

### Essential oils analysis

For the first time, a comparative study has been done between the essential oils of *Rhus coriaria* var. *zebaria*. According to the GC–MS analysis, 96.66% of total compositions were identified in Akre accession. 2,4-Decadienal (14.92%), caryophyllene oxide (12.9%), E-caryophyllene (8.41%) and cembrene (4.46%) were the major components the essential oil compositions of *Rhus coriaria* var. *zebaria* accessions (Table 1). About Dostic sumac, 94.17% of total oil compositions were identified and caryophyllene oxide (23.1%), 2,4-decadienal (13.5%), E-caryophyllene (5.66%), and octanoic acid (3.14%) were the main compounds. 95.09% of total oil for sumac that collected from Xasto Zheri were identified. Z, E,2,13-octadecadien-1-ol (45%), caryophyllene oxide (14.3%), nonanoic acid (12.4%) and E-caryophyllene (6.56%) were the major components. 97.81% of the components of Hashtka sumac essential oil were identified. According the result, E-caryophyllene (34.5%), cembrene (10.01%), di-(2-ethylhexyl) phthalate (6.16%), and valencene (5.32%) were the main compounds. About Kavilca sumac, 87.17% of the compounds were identified and caryophyllene oxide (15.2%), 2,4-decadienal (9.11%), cembrene (9.04%), and E-caryophyllene (6%) were major compounds (Table 1).

**Table 1:** Essential oils compounds (%) of *Rhus coriaria* var. *zebaria* accessions.

Compounds (%)	Akre	Dostic	Xasto Zheri	Hashtka	Kavilca
n-Heptanal	0.3	–	–	–	–
α.-Pinene	0.24	–	0.95	0.07	–
1-Heptanol	0.27	–	–	–	–
1-Octen-3-ol	0.23	–	–	–	–
Furan	0.28	–	–	0.14	0.07
Decane	1.78	–	0.87	2.2	1
Octanal	1.23	0.75	1.33	0.32	0.5
2-Octenal	0.9	–	0.41	–	–
1-Octanol	1.21	–	–	–	–
Heptanoic acid	0.46	–	–	–	–
Nonanal	2.33	1.59	1.83	3.45	2
2-Nonenal	0.45	–	–	–	–
4-Dodecene	0.17	–	–	–	–
Octanoic acid	0.97	3.14	–	–	3.06
Decanal	1.03	0.85	0.81	0.32	0.67
2,4-Nonadienal	0.15	–	–	–	–
α.-Caryophyllene	0.23	–	–	–	–
E-2-Tridecenal	2.64	2.91	–	–	2.08
α.-Fenchyl acetate	0.21	–	–	–	–
2,4-Decadienal	14.92	13.5	4.17	0.47	9.11
Nonanoic acid	1.09	2.64	12.4	0.21	1.5
(E)-1-(methoxymethoxy)-1-tetradecen-3-ol	2.81	–	–	–	–
Tetratriacontane	0.27	–	–	–	–
Trichloroacetic acid	0.25	–	–	–	–
Decanoic acid	1.16	1.08	1.68	–	1
Isocaryophyllene	0.46	0.31	0.19	1.12	0.5
E-Geranyl acetone	0.63	–	–	–	–
α.-Humulene	1.32	–	–	–	–
2-Cyclopenten-1-one,2-pentyl-	0.36	0.51	0.69	–	0.34
Naphthalene	0.34	0.5	0.27	0.5	0.45
Undecanoic acid	0.28	–	–	–	–
3-(3-Butenyl)cyclohexanone	0.3	–	–	–	–
β.-Selinene	0.32	0.47	0.37	–	0.3
α.-Selinene	0.48	0.33	–	–	0.35
Camphor	0.14	–	–	–	–
α.-Farnesene	0.7	–	–	–	–
(1-S,Z) Calamenene	0.31	–	–	–	–
(-)-Isopulegol	0.92	–	–	–	–
ɣ.-Selinene	0.19	–	0.27	–	–
7-Epi-.α.-selinene	0.3	–	–	–	0.07
Caryophyllene oxide	12.9	23.1	14.3	1.2	15.2
Dodecanoic acid	0.25	–	–	–	–
Caryophyllenyl alcohol	1.63	–	–	–	0.31
Z-.α.-Bisabolene	0.39	–	–	–	–
Veridiflorol	0.72	–	1.16	–	–
Humulene oxide	1.57	2.11	1.41	–	1.9
E-Caryophyllene	8.41	5.66	6.56	34.5	6
Δ.-Selinene	1.4	–	–	–	–
α.-Caryophylladienol	1.21	–	–	–	–
Azulene	0.67	–	–	–	0.1
2-Naphthalenemethanol	0.43	1.8	3.13	0.71	1.7
Caryophylla-3,8(13)-dien-5.β.-ol	3	–	–	–	–
Tetradecanoic acid	0.79	–	–	–	–
Octadecane	0.28	0.77	0.63	0.23	0.2
Hexahydrofarnesyl acetone	0.45	0.85	1.02	0.28	0.5
1,2-Benzenedicarboxylic acid, bis(trimethylsilyl)ester	0.35	–	0.96	–	–
Hexadecanoic acid	0.96	–	–	0.31	0.22
Oxirane	0.24	0.36	–	–	0.12
2-Heptadecanone	0.56	0.96	1.09	–	0.81
Farnesyl acetone	1	1.5	1.77	0.18	0.99
Cembrene	4.46	3.1	4.85	10.01	9.04
β.-Elemene	2.12	–	2.24	–	–
Z-Salvene	1.2	–	–	–	–
Thunbergol	0.44	–	–	–	–
Longifolenaldehyde	0.25	0.45	–	–	0.54
2-Pentenoic acid	0.41	0.64	1.15	–	0.52
Labda-8(17),13E-dien-15-al	3.4	0.36	0.74	–	–
2-Methyl-3-(3-methyl-but-2-enyl)-2,3 epoxy-1,4-naphthoquinone	0.32	1.94	–	–	1.3
Stearic acid	0.38	–	–	–	–
Nonadecane-2,4-dione	0.21	–	–	0.1	0.03
3-Cyclopentylpropionic acid, but-3-yn-2-yl ester	1.13	–	–	–	–
2,2-Dimethyl-3-(3,7,16,20-tetramethyl-heneicosa-3,7,11,15,19-pentaenyl)-oxirane	1.03	–	–	–	–
Caryophyllene	0.91	–	–	–	–
Di-(2-Ethylhexyl)phthalate	0.56	–	–	6.16	3
Cyclopropane, pentyl	–	0.69	0.67	–	0.4
E-2-Decenal	–	2.27	1.49	0.45	1.8
2-Methylene cyclopentanol	–	0.45	–	–	0.32
Tetradecane	–	0.35	0.53	0.89	0.5
Humulen-(v1)	–	0.38	0.46	1.57	0.77
Valencene	–	1.18	–	5.32	1.01
1,4,7,-Cycloundecatriene	–	1.09	1.11	5.19	0.9
(Z,Z)-.α.-Farnesene	–	1.21	–	–	1
Spiro[5.6]dodecane-1,7-dione	–	1.5	–	–	0.79
1H-Cycloprop[e]azulene, decahydro-1,1,4,7-tetramethyl-	–	0.13	0.31	0.9	0.2
E-Z-.α.-Bisabolene	–	0.61	–	–	0.53
α.-Caryophyllene alcohol	–	2.34	2.62	2.37	2.5
(-)-Caryophyllene oxide	–	2.14	–	0.7	1.5
Cyclopropane carboxamide	–	0.65	–	–	0.3
2H-Inden-2-one, 1,4,5,6,7,7a-hexahydro-5-hydroxy-7,7-dimethyl-	–	1.55	0.28	–	1
2,7-Octadiene, 1-butoxy-	–	1.06	–	–	0.8
Z,E-2,13-Octadecadien-1-ol	–	0.2	45	–	–
Nonadecane	–	0.17	0.23	–	–
Eicosane	–	2.26	0.32	–	2.05
Thujone	–	0.83	–	–	0.5
Agatholic acid	–	0.93	–	–	0.6
Tridecane	–	–	4.06	0.25	0.1
Bicyclo[2.2.1]heptane-2,5-dione	–	–	0.34	1.42	0.6
1,2-Dihydropyridine	–	–	0.41	–	–
β.-Sesquiphellandrene	–	–	0.55	–	–
α.-Amorphene	–	–	0.53	–	–
4-methyl-exo-tricyclo[6.2.1.0(2.7)]undecane	–	–	1.44	–	–
( ±)-Anastrephin	–	–	1.13	–	–
d-Nerolidol	–	–	0.62	–	–
Isoaromadendrene epoxide	–	–	1.42	–	–
Norsesterterpene dien ester	–	–	1.72	–	–
1H-Imidazole-4-carboxaldehyde	–	–	0.59	–	–
Heptadecane	–	–	1	0.11	0.2
Tridodecylamine	–	–	0.34	–	–
1,5,9-Cyclotetradecatriene	–	–	2.7	1.67	0.7
Tetrahydroxy cyclopentadienone	–	–	0.48	–	–
8-Hexadecyne	–	–	2.04	–	–
Thymol	–	–	–	0.61	0.2
5-Methylnonane	–	–	–	0.1	–
3-Methylnonane	–	–	–	0.9	0.3
1-Tetradecanol	–	–	–	0.39	–
Dl-Limonene	–	–	–	0.18	–
Z-Ocimene	–	–	–	0.22	–
1,3,6-Octatriene	–	–	–	0.31	0.1
Cyclohexene	–	–	–	0.22	–
Undecane	–	–	–	0.82	0.5
Linanol	–	–	–	0.23	–
Dodecane	–	–	–	1.3	0.32
Vitispirane	–	–	–	0.27	–
Undecanal	–	–	–	0.58	0.2
α.-Copaene	–	–	–	0.17	–
Pentadecane	–	–	–	0.44	0.1
Heneicosane	–	–	–	0.55	0.2
2-Pentacosanone	–	–	–	0.3	–
Bicyclo[4.1.0]hept-2-ene	–	–	–	0.54	–
8,11-Octadecadienoic acid, methyl ester	–	–	–	0.68	0.3
Oleic acid	–	–	–	0.4	0.1
Linaloic acid	–	–	–	0.23	–
3-Heptanone, 6-(dimethylamino)- 4,4-diphenyl-	–	–	–	5.05	0.3
Total	96.66	94.17	95.09	97.81	87.17

The main compounds between the essential oils of accessions were Z, E-2,13-octadecadien, caryophyllene oxide, 2,4-decadienal, E-caryophyllene and cembrene (Table 1). Cemberene is a natural diterpenoid found in terrestrial and marine organisms. Cerbenoid has been found to have inhibitory activity against tumor promoters and microbial and/or viral infections^27^. Caryophyllene oxide is a sesquiterpene that is used in cosmetics, medicines, as a preservative in the food industry and in the training of drug detection dogs. It has anti-fungal, insecticidal and anti-platelet properties^28^. (E, E)-2,4-decadienal is an aromatic substance that is used in the perfumery industry and in the food industry as a flavoring, but due to its possible carcinogenicity, its use has terms and restrictions^29^. Trans-caryophyllene is one of the important components of essential oils of several plant species. Especially β-caryophyllene, which is the main sesquiterpene of some herbal essential oils and has medicinal activities such as anti-cancer, anti-inflammatory, antimicrobial, heart and liver protection, etc^30^.

According to a study on its extract in Duhok, Iraq, by Mohammed et al.^25^, it has been determined that it contains compounds such as chlorogenic acid, gallic acid, caffeic acid and benzoic acid. A study about the essential oil of fruit pericarp in Turkey, shows that the predominant compounds were limonene, nonanal and (Z)-2-decenal^31^. Another study shows that (E)-caryophyllene, n-nonanal, cembrene, α-pinene, (2E, 4E)-decadienal and nonanoic acid were the mein compounds in Iran^4^. Our research result, shows that Iraqi sumac is similar to Iranian sumac in terms of essential oil composition.

### Total phenolic content (TPC) analysis

**Figure 1:**
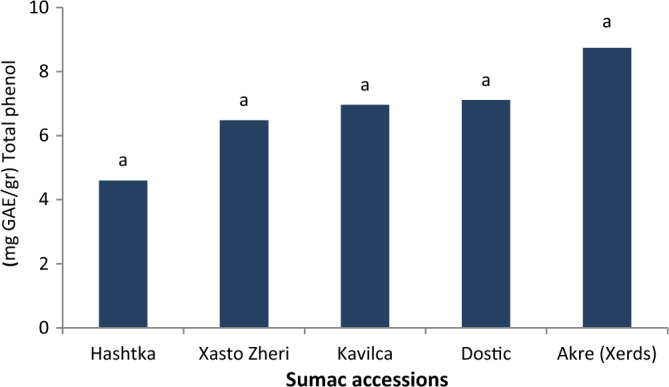
Comparison of mean total phenol in different sumac accessions.

*Rhus coriaria* var. *zebaria* which collected from Akre (Xerds) with 8.74 mg g-1DW had the highest amount of TPC compared with other regions. After that, the accession of Dostic region with 7.11 mg GAE/g had more total phenol. The lowest amount of phenolic compounds was belonging to the Hashtka region with 4.6 mg GAE/g however, they had no significant difference (Fig. 1 & Table 2). Previous study on brown and red sumac, showed that, the phenolic content of brown sumac extract was higher than red sumac^32^. Another study, showed that aqueous extract of sumac collected from Diyarbakir Cermik has more TPC than Mardin's sumac^33^.

**Table 2:** Total flavonoids and phenols of sumac extract in the different areas and harvesting time.

Area	Harvest time	Phenolic (mg GAE/100 g extract)	Flavonoids (mg QE/g extract)
Xasto Zheri	17/8/2020	6.48	4.15^d^
Akre (xerds)	7/8/2020	8.74	6.05^a^
Dostic	13/8/2020	7.11	5.66^b^
Kavilca	13/8/2020	6.96	5.05^c^
Hashtka	3/8/2020	4.62	3.05^e^

The ANOVA procedure tests (LSD) for phenol and flavonoids means with the same letter are not significantly different.

### Total flavonoid content (TFC)

**Figure 2:**
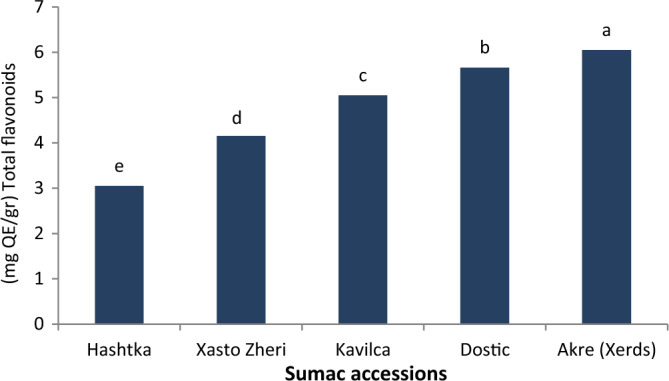
Comparison of mean total flavonoid in different sumac accessions.

*Rhus coriaria* var. *zebaria* which collected from Akre (Xerds) with 6.05 mg QE/g TFC had the higher amounts of flavonoids than the other locations. After that, the sumac of Dostic with 5.66, Kavilca with 5.05, and Xasto Zheri with 4.15 mg QE/g had the highest amount of flavonoid, respectively. The lowest flavonoid content with 3.05 mg QE/g was observed in Hashtka sumac. They were significantly different (Fig. 2 & Table 2). Previous study on aqueous extract of sumac in Turkey, showed that, Diyarbakir Cermic's sumac has more TFC than Mardin's^33^.

### Total anthocyanin content of the extracts

The highest content of anthocyanin was found in sumac of Hashtka (25.57 ± 0.13 mg CyE/g DW) and Akre (Xerds) (25.48 ± 0.12 mg CyE/g DW). After them, sumac of Xasto Zheri with 10.53 ± 0.5 and Dostic with 8.96 ± 0.11 mg CyE/g DW had the highest amount of antocyanins. Sumac of Kavilca with 2.516 ± 0.10 mg CyE/g DW had the lowest antocyanin content (Table 3).

**Table 3:** Total anthocyanin content of sumac extract in the different areas and harvesting time.

Area	Harvest time	Anthocyanin (mg cyanidin-3-glucoside equivalent/g extract)
Xasto Zheri	17/8/2020	10.539 ± 0.5
Akre (xerds)	7/8/2020	25.48 ± 0.12
Dostic	13/8/2020	8.968 ± 0.11
Kavilca	13/8/2020	2.516 ± 0.10
Hashtka	3/8/2020	25.57 ± 0.13

### Antioxidant activity (DPPH)

All extracts had radical scavenging effects at all concentrations. Although only the radical scavenging effect of the Dostic sumac extract was slightly better than ascorbic acid effect compared with other extracts at a concentration of 400 µg/mL. In general, the average antioxidant activity of sumac extract in Hashtka with 74.41 µg/mL was better than other regions. After that, Dostic sumac with 78.76 and Akre Xerds sumac with 79.43, and Kavilca sumac with 87.02 µg.mL had more activity. Xasto sumac with 87.79 µ/mL had the least activity (Table 4).

**Table 4:** Radical scavenging activity of sumac (*Rhus coriaria* var. *zebaria*) fruit extract.

Location	Plant extracts, methanol concentrations	IC_50_ µg.mL	AA (mg AAE/g DW)
Xasto	50	94.736^B-A^	92.72^B-C-D^
	100	92.1^B-E-C-D^	89.09^F-E-C-D^
	200	88.17^F-E-G-D^	83.6^J-I-G-H^
	400	85^F-I-G-H^	79.83^J-K^
	800	78.94^C^	70.90^O-M-N^
Kavilca	50	95.64^B-A^	93.95^B-C^
	100	93.86^B-C^	91.50^B-E-C-D^
	200	90.79^B-E-C-D^	87.27^F-E-G-H^
	400	82.45^J-I^	79.83^J-K^
	800	72.36^L-M-N^	70.89^O-M-N^
Akre xerds	50	69.2^O-P-N^	57.57^R-Q^
	100	90.7^B-E-C-D^	87.27^F-E-G-H^
	200	91.24^B-E-C-D^	87.8^F-E-G-H^
	400	79.8^C-B-J-K^	72.12^L-M-N^
	800	66.22^O-P^	53.33^R^
Hashtka	50	87.7^F-E-G-H^	83.03^J-I-H^
	100	86.13^F-I-G-H^	79.39^J-K^
	200	74.56^L-M^	64.84^P^
	400	66.22^O-P^	53.33^R^
	800	57.45^R-Q^	41.25^S^
Dostic	50	76.38^L-K^	67.27^O-P^
	100	68.85^O-P-N^	56.97^R-Q^
	200	60.52^Q^	45.47^S^
	400	95.57^B-A^	98.88^A^
	800	92.77^B-C-D^	41.21^S^

Means followed by the same letter for each treatment do not differ significantly from each other’s according to Duncan’s multiple range test at 5% level.

### Total acidity (%)

The result showed that sumac collected from Dostic at an altitude of 1154 m had the highest acidity (5.2 g/L). After that sumac of Kavilca with 4.6, Hashtka with 4.3 and Xasto Zheri with 3.9 g/L had more acidity. The accession belonging to Akre (Xerds) had the lowest acidity. The results of the study conducted by Sakhr and El Khatib on Syrian sumac are similar to these results^34^.

### Total pH concentration in sumac fruit

*Rhus coriaria* var. *zebaria* collected from Xasto Zheri, which was the highest region (1212 m), with 2.867 had the highest pH. Then, Akre (Xerds) with 2.819, Hashtka with 2.792 and Dostic sumac with 2.79 had the higher pH. Kavilca's sumac with 2.675 had the lowest pH. So, the sourest taste belongs to sumac of Kavilca region. According to a study on Iranian sumac, it was found that they have a slightly lower pH than the sumac of our research areas^35^. Another study showed that sumac of Mersin (Turkey) had higher pH than Iraqi accessions^36^.

### Fruit minerals content

Nitrogen (N2) %: The highest amount of N2 was found in Hashtka accessions (1.54%), followed by Xasto Zheri (1.26%). After them, Dostic (0.84%) and Kavilca accessions (0.64%) had more N2. The accession belonging to Akre (Xerds) had the lowest amount of N2 (0.44%). In a study on some species of *Rhus*, it was found that poisonous species had more phosphorus and nitrogen than nonpoisonous species^37^ (Table 5).

**Table 5:** Minerals of sumac in different areas (mg.50 g).

Area	Xasto Zheri	Akre (xerds)	Dostic	Kavilca	Hashtka
Nitrogen (N_2_)	1.26	0.44	0.84	0.64	1.54
Phosphorus (P)	666.66	692	358.97	333.33	333.45
Potassium (K)	5537.09	4798.81	5721.66	5352.52	8235.36
Calcium (Ca)	2200	1830	2080	1400	2040
Magnesium (Mg)	1320	1080	1248	840	1224
Sodium (Na)	795.75	707.33	884.17	972.59	176.83
Chloric (Cl)	213	284	288.61	213	195.25

#### Phosphorus (P)

There was the most P in sumac accession collected from Akre (Xerds) (692 mg/50 g). Then, Xasto Zheri's sumac with 666.66 and Dostic's sumac with 358.97 mg/50 g had more P. The lowest amount of P with a slight difference was found in the accessions collected from Hshtka (333.45 mg/50 g) and Kavilca (333.33 mg/50 g) (Table 5). Previous study by Abdulqader Amin^38^ showed that ethanol extract of sumac in Kurdistan region of Iraq contained 109.88 mg/100 g P, which was low compared with our results. Another study showed that one kg of Syrian sumac has a P content close to this variety (327.70 mg/Kg), but the phosphorus in one kg of Chinese sumac is much higher (1032 mg/Kg)^39^.

#### Potassium (K)

Hshtka's sumac with 8235.36 mg/50gr had the highest K. After that, Dostic's sumac with 5721.66, Xasto Zheri's sumac with 5537.09 mg/50gr and Kavilca's sumac had more K. The lowest amount of K was found in Akre's sumac (4798.81 mg/50 g) (Table 5). According to the results of this study and previous studies, it was found that potassium is the most mineral in sumac^38,39^.

#### Calcium (Ca)

The most Ca was found in the accession of Xasto Zheri region (2200 mg/50 g). Then, accessions of Dostic, Hashtka and Akre (Xerds) respectively had more Ca (2080, 2040 and 1830 mg/50 g). The lowest amount of K belonged to Kavilca region (1400 mg/50 g) (Table 5). In this study and many previous studies, after K, Ca is the second most abundant mineral in sumac^38,39^.

#### Magnesium (Mg)

The amount of Mg in sumac of Xasto Zheri region was higher compared to other regions (1320 mg/50 g). After that, sumac of Dostic, Hashtka and Akre (Xerds) respectively had more Mg (1248, 1224 and 1080 mg/50 g). Sumac of Kalvica with 840 mg/50 g had the lowest Mg (Table 5). According to a study on Turkish sumac, it was found that the amount of Mg in our samples is higher than theirs^6^.

#### Chloride (Cl)

The sumac of Dostic (288.61 mg/50 g) and Akre (Xerds) (284.4 mg/50 g) regions had the highest Cl. Then, sumac of Kavilca with 213.8 and Xasto Zheri with 213 mg/50 g had more Cl. The sumac of Hashtka region with 195.25 mg/50 g had the least Cl (Table 5).

#### Sodium (Na)

Sumac collected from Kavilca with 972.59 mg/50 g had the most Na. Then, respectively, the accessions belonging to Dostic, Xasto Zheri and Akre (Xerds) had more Na (884.17, 795.75 and 707.33 mg/50 g). The sumac of Hashtka region had the lowest Na by a large margin compared to other regions (176.82 mg/50 g) (Table 5). Compared to previous studies on Chinese, Turkish and Iraqi Kurdestan, our plants had more minerals.

### Total crude oil and wax (%)

**Figure 3:**
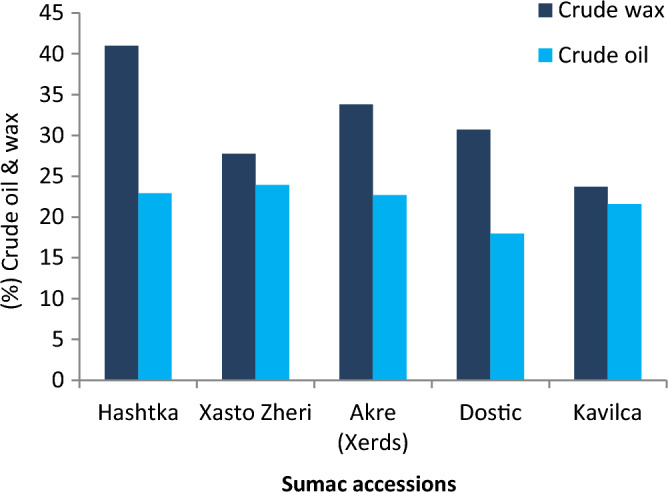
Comparison of crude oil and wax in different sumac accessions.

The *Rhus coriaria* var. *zebaria* oil that was extracted had a mild odor, the black-browned or deep yellow in color and acidity taste and it was quite viscous at room temperature. Hashtka's sumac with 40.99% had the crudest oil. After that, sumac of Akre (Xerds) with 33.79%, Dostic with 30.71% and Xasto Zheri with 27.75% had more crude oil. Kavilca’s sumac with 23.72% had the lowest crude oil (Fig. 3). A study showed that Turkish sumac (*Rhus coriaria*) had 7.4% oil and crude oil of fresh sumac is 10.6%^37^.

Sumac of Xasto Zheri region with 23.94% had the crudest wax. Then, accessions of Hashtka with 22.9%, Akre (Xerds) with 22.69% and Kavlica with 21.59% had more crude wax. The accession belonging to Dostic with 17.98% had the least amount of crude wax (Fig. 3).

### Total protein in sumac fruit

*Rhus coriaria* var. *zebaria* collected from Hashtka had the highest percentage of protein (9.6%). Then, respectively, the percentage of protein in sumac collected from Xasto Zheri (7.8%), Dostic (5.25%) and Kavilca (4%) was higher. The lowest percentage of proten was found in sumac of Akre (Xerds) (2.8%).

### Total carbohydrate (%)

The sumac collected from Xasto Zheri had by far the highest percentage of carbohydrates (17.8%). After that, the accessions of Dostic, Hashtka and Kavilca respectively had more carbohydrates (11.22, 8.78 and 7.32%). The lowest percentage of carbohydrates belonged to Akre (Xerds) accession (1.95%). A previous study on *Rhus typhina* L. showed that its carbohydrate content is 22.1%^40^.

### Antibacterial properties of sumac oil

The most antibacterial property against *Staphylococcus* was found in sumac oil of Xasto Zheri region (20.4 ± 3.3 mm). Its inhibitory effect was better than sumac oil and extract from other regions and even better than the antibiotics used in the experiment. After that, sumac oil collected from Dostic (with 18.14 ± 2.4 mm) and Akre (Xerds) (with 16 ± 2.2 mm) had more antibacterial properties that had a better effect than penicillin and amoxicillin. The inhibitory rate of sumac oil of Hashtka and Kavilca regions were close to the same (14.21 ± 1.7 and 14 ± 2.6 mm) and were better than penicillin. Also, the most antibacterial property against *E. coli* was found in sumac oil of Dostic (15 mm) that had a better effect than florfenicol. After that, sumac oil collected from Hashtka, Kavilca and Xasto Zheri respectively had better antibacterial properties (13, 12 ± 3.7 and 10 mm), but none of them had a better effect than antibiotics. The lowest inhibitory effect of sumac oil against *E. coli* was from Akre region (6.32 ± 1.3 mm) (Table 6).

**Table 6:** Inhibition zone diameter of *Rhus coriaria* var. *zebaria* oil and water extract on dental caries pathogens (mm).

Region	Bacterial strain	Water extract	Sumac oil	Penicillin	Enrofloxacin	Amoxicillin	Florfenicol
Akre	*E. Coli*	–	6.32 ± 1.3	21.4 ± 4.3	26 ± 2.1	18.2 ± 3.4	13.1 ± 2.4
	*Staph*	17 ± 3.6	16 ± 2.2	13.2 ± 3.2	19 ± 1.5	15 ± 2.1	20 ± 1.6
Xasto Zheri	*E. Coli*	–	10	21.4 ± 4.3	26 ± 2.1	18.2 ± 3.4	13.1 ± 2.4
	*Staph*	15 ± 2.3	20.4 ± 3.3	13.2 ± 3.2	19 ± 1.5	15 ± 2.1	20 ± 1.6
Hashtka	*E. Coli*	–	13	21.4 ± 4.3	26 ± 2.1	18.2 ± 3.4	13.1 ± 2.4
	*Staph*	8 ± 4.2	14.21 ± 1.7	13.2 ± 3.2	19 ± 1.5	15 ± 2.1	20 ± 1.6
Dostic	*E. Coli*	–	15	21.4 ± 4.3	26 ± 2.1	18.2 ± 3.4	13.1 ± 2.4
	*Staph*	12 ± 3.5	18.14 ± 2.4	13.2 ± 3.2	19 ± 1.5	15 ± 2.1	20 ± 1.6
Kavilca	*E. Coli*	–	12 ± 3.7	21.4 ± 4.3	26 ± 2.1	18.2 ± 3.4	13.1 ± 2.4
	*Staph*	12 ± 2.8	14 ± 2.6	13.2 ± 3.2	19 ± 1.5	15 ± 2.1	20 ± 1.6

None of the water extracts had any effect against *E. coli*, but against *Staph*, Akre had the greatest inhibition (17 ± 3.6 mm), which worked better than the sumac oil of the same region, penicillin and amoxicillin. After that, water extract of sumac collected from Xasto Zheri had more inhibition that was more effective than penicillin (15 ± 2.3 mm). The inhibitory effect of the rest belonged to Dostic, Kavilca and Hashtka, respectively (12 ± 3.5, 12 ± 2.8 and 8 ± 4.2 mm), none of which worked better than antibiotics (Table 6).

A previous study on ethanolic extract of Iranian sumac showed a strong antimicrobial activity for Gram positive and Gram negative bacteria^41^. Another study on water extract of sumac showed that to be effective against all the test organisms with Gram positive strains being more sensitive than Gram negative strains^42^.

## Conclusions

There was not difference between the essential oil yields of *Rhus coriaria* var. *zebaria* accessions. Comparing the essential oil of this variety of Iraqi sumac with the essential oil of Turkish and Iranian sumac, it was found that it is more similar to Iranian sumac. None of the water extracts had any effect on *E. coli* bacteria. The differences in different characteristics can be caused by the height of the place where the plants are collected and their environmental factors. In general, the difference in results can be caused by genetic variation. In most cases of evaluation, the accession belonging to Kavilca was weaker and had less medicinal value; but Xasto Zheri's accession was the best in most cases and it is worth preserving and cultivating. However, depending on the results, they can be used and cultivated for different purposes.

## Data Availability

All the data generated or analyzed during the current study were included in the manuscript. The raw data is available from the corresponding author on reasonable request.

## Abbreviations

GC–MS: Gas chromatography–mass spectrometry
DPPH: 2,2-Diphenyl-1-picrylhydrazyl
